# Fluoride-Induced Microhardness Changes in Resin-Modified Glass Ionomer Cements: A Comparative Study

**DOI:** 10.4317/jced.62349

**Published:** 2025-01-01

**Authors:** Katherine Susan Rufasto-Goche, ES Cerro-Olivares, NF San Martín-Hilario, Flor M. Santander-Rengifo, Alexis G. Murillo-Carrasco, María Victoria Lizarbe-Castro

**Affiliations:** 1 Faculty of Dentistry, Universidad Nacional Federico Villarreal, Lima, Peru; 2 Postgraduate School, Universidad Nacional Federico Villarreal, Lima, Peru; 3 Postgraduate School, Universidad Privada San Juan Bautista, Lima, Peru; 4 Academic Program of Dentistry, Universidad Peruana de Ciencias Aplicadas, Lima, Peru; 5 Immunology and Cancer Research Group (IMMUCA), Lima, Peru

## Abstract

**Background:**

Resin-modified glass ionomer cement (RM-GIC) is widely used in clinical dental procedures as a restorative material due to its chemical composition. It is known for its strong adhesion to dental structures and its fluoride content. However, fluoride in RM-GIC is insufficient for preventing the formation of carious lesions, making the use of fluoride gel and varnish necessary as preventive strategies. Nevertheless, there may be adverse interactions between RM-GIC and fluoride, which could compromise the properties of these restorative materials. Therefore, it is crucial to understand the physicochemical and biological properties of the products used in dental treatments. This experimental study aimed to evaluate the effect of the following fluorides: 2% neutral sodium fluoride (NaF), 1.23% acidulated phosphate fluoride (APF), and 0.1% fluoride varnish (7700 ppm F) in the mi-cro-hardness of the RM-GIC.

**Material and Methods:**

Using GC Fuji II LC-A2, 80 RM-GIC discs measuring 6cm x 4cm were made and immersed in artificial saliva for seven days. Then, the discs were washed, dried, and randomly divided into four groups, and the initial surface microhardness was measured. After that, the discs were immersed in the three fluorides to measure the microhardness for a second time. The average value of the surface microhardness of the RM-GIC in the final phase (exposure to fluorides) of the three experimental study groups is lower than the initial phase (non-exposure to fluorides).

**Results:**

There was a significant decrease in the microhardness of the ionomer with the application of the three fluorides (*p*= 3.6x10-12). Particularly, the treatment with 1.23% acidulated phosphate fluoride demonstrated higher variation than 2% neutral sodium fluoride (*p*=0.0063) or 0.1% fluoride varnish (*p*=2.2x10-5).

**Conclusions:**

2% neutral sodium fluoride, 1.23% acidulated phosphate fluoride, and 0.1% fluoride varnish (7700 ppm F) applied to RM-GIC decreases surface microhardness.

** Key words:**Acidulated fluorine phosphate; fluoride treatment; fluorine varnish; glass ionomer cement; sodium fluoride; surface micro-hardness.

## Introduction

Dental caries is one of the most prevalent diseases worldwide, affecting both children and adults. Due to the association between carious lesions and fluoride-containing restorative cements, interest has grown in the development of restorative materials, coating cements, sealants, and orthodontic cements. Fluoride, as an anti-cariogenic agent, operates through various mechanisms, such as reducing demineralization, enhancing re-mineralization, inhibiting dental plaque formation, and suppressing microbial growth and metabolism (1,2). Topical fluorides are applied directly to the teeth to prevent dental caries (3).

In the quest for a fluoride-releasing restorative material with optimal physical and chemical properties, glass ionomer cement (GIC) has become a popular choice in pediatric dentistry. This is due to its biocompatibility, bi-oactivity, adhesion to dental structures, ease of handling, extended fluoride release, and favorable biomimetic properties (4,5). GIC offers a unique advantage in its ability to modify its biological and physical characteristics, which is essential for precise control during manipulation and clinical application. Understanding its chemical, physical, and biological properties is key to maximizing its clinical benefits (6).

The introduction of resin-modified glass ionomer cement (RM-GIC) marked a significant advancement in dentistry (7,8) due to its improved mechanical properties, increased resistance to wear and fracture, and reduced sensitivity to moisture compared to conventional GIC (5,9,10). RM-GIC can also be reactivated through successive exposures to external fluoride sources, such as fluoride mouthwash or toothpaste, which provides a long-term bacteriostatic effect (11-14).

Long-lasting restorative materials require high surface hardness, which is one of the most important physical properties for dental materials. Surface hardness correlates with resistance to both compression and abrasion (9,15). RM-GIC can absorb more fluoride ions, acting as a rechargeable fluoride delivery system when exposed to topical fluoride treatments such as neutral sodium fluoride gel, acidulated phosphate fluoride, or fluoride varnish (9,16-19). However, the high reactivity of fluorinated compounds used in these topical treatments, particularly those applied in dental offices, may deteriorate the aesthetic properties of restorative materials, negatively im-pacting their clinical durability (9,20-22). Additionally, these compounds may reduce the mechanical properties of RM-GIC (23), leading to surface erosion and degradation (24).

Fuji II LC-GC Glass Ionomer Light-cured Universal Restorative (2LC, https://www.gc.dental/) is one of the most widely used resin-modified glass ionomer cement (RM-GIC) in clinical dentistry. Its chemical composition includes fluoroaluminosilicate glass, polyalkenoic acid, HEMA, aluminum chloride, and camphorquinone. It is employed as a final restorative material, luting cement, lining cement, and as a pit and fissure sealant. RM-GICs are particularly valuable when long-term adhesion is required, such as in non-carious cervical lesions. Recommended indications include Class III and V restorations, particularly for cervical erosions and root surface caries, restoration of primary teeth, core build-ups, cases re-quiring radiopacity, geriatric applications, and as a base or liner. However, given the limited scientific research on this dental product, it was decided to analyze the effect of fluoride on one of the representative restorative materials in glass ionomer cement, Fuji II LC.

This study aimed to evaluate the effect of 2% neutral sodium fluoride, 1.23% acidulated phosphate fluoride, and 0.1% fluoride varnish (7700 ppm F) on the surface microhardness of RM-GIC. The null hypothesis was that there would be no significant differences in surface microhardness when RM-GIC was immersed in these fluoride solutions.

## Material and Methods

-Type of study and sampling planning

This experimental, *in vitro*, and analytical study was conducted at the Dental Materials Laboratory of the Universidad Peruana Cayetano Heredia (UPCH), as well as the High Technology Laboratory Certificate (ISO/IEC 17025) in Lima, Peru, under approving resolution No. 1529-2023-CU-UNFV. The study followed standardized guidelines for *in vitro* experimental dental research, in accordance with ISO 4049, which is used for polymeric materials (25).

-Sample size calculation and selection criteria

The study sample consisted of 80 test specimens made with RM-GIC (GC Fuji II LC®-Powder/Liquid) (26-28). A pilot study was conducted using 36 discs (nine per group: control group, group exposed to 2% neutral sodium fluoride, group exposed to 1.23% acidulated phosphate fluoride, and group exposed to 0.1% fluoride varnish) to evaluate the material samples. These were not included in the final statistical analysis. The pilot study served as a basis for calculating the total sample size, utilizing IBM SPSS version 29 software (variance analysis), with consideration of confidence interval (CI=95%), statistical power (90%), and group ratio (1:1) for the three experimental groups and one control group. Values of mean and standard deviation for each group were obtained from the pilot study: Group 1 (4.0±2.1), Group 2 (6.3±2.6), Group 3 (1.7±1.5), and Group 4 (-0.8±2.7). Microhardness data were used for sample size calculation, determining the final sample size in 18 discs per group.

-Preparation of the sample and microhardness test

**Figure 1:**
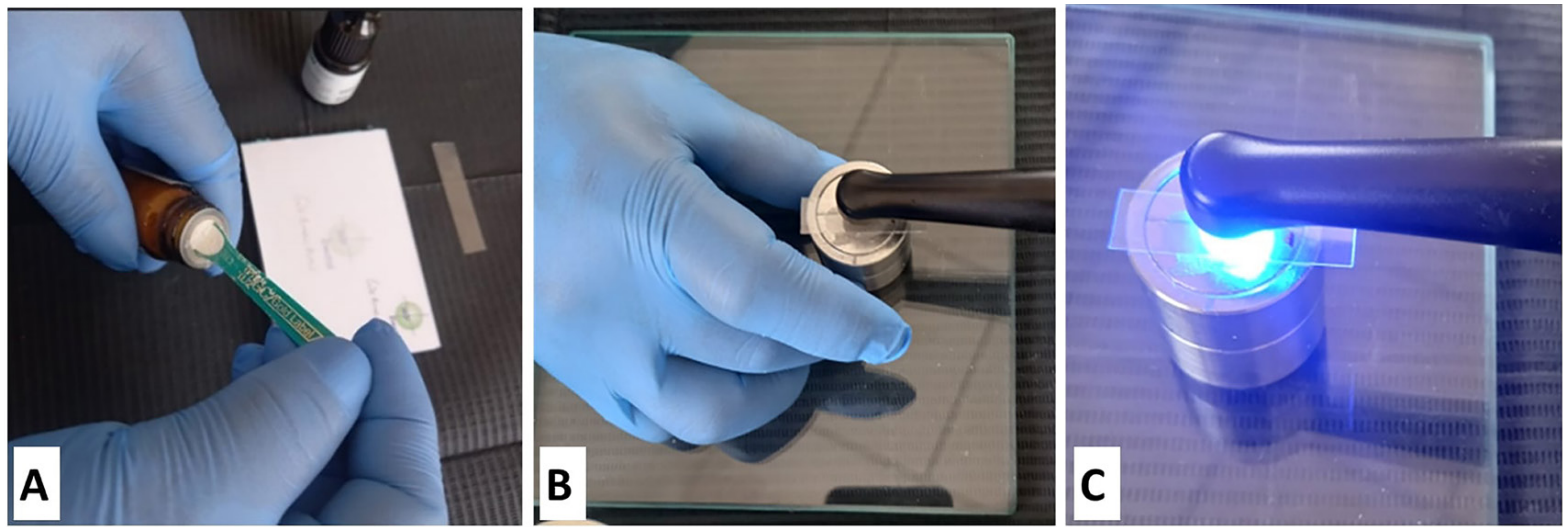
Preparation of RM-GIC discs. (A) The powder and liquid components of RM-GIC were mixed to initiate the formation of polyacid. (B) The mixture was placed into a steel mold with dimensions of 6 mm in depth and 4 mm in diameter, manufactured according to ISO 4049:2009 specifications. (C) The cement was photoactivated for 20 seconds using a light intensity of 1000 mW/cm².

First, a plastic spatula was used to mix the powder and liquid components of RM-GIC, Fuji II LC-GC Glass Ionomer Light-cured Universal Restorative 2LC, provided by GC Corporation, Tokyo, Japan (Fig. 1A), in accordance with the manufacturer’s instructions. Most available forms consist of glass powder and separate liquid polyacid components. The polymerization of the polymer in an aqueous solution occurs via free radicals, resulting in the formation of polyacid. The mixture was then placed in a steel mold (29) with a depth of 6 mm and a diameter of 4 mm (Fig. 1B), manufactured according to ISO 4049:2009 specifications to create standardized specimens.

To ensure smooth and uniform surfaces, a transparent matrix strip (30) and a glass platen were used to apply pressure. The cement was photoactivated for 20 seconds from the top of the mold using a VALO™ Grand Cordless LED lamp (Ultradent, South Jordan, USA) at a power of 1000 mW/cm², as recommended by the manufacturer (Fig. 1C) (31). The intensity of the light was verified with a Bluephase®Meter II dental radiometer (Ivoclar © Vivadent, Schaan, Liechtenstein).

Samples were stored in bottles containing 20 ml of salivary solution in a 100% humidity environment at 37°C (32). This commercial solution contains the following components: 0.084 g of sodium chloride, 0.120 g of potassium chloride, 0.015 g of calcium chloride dihydrate, 0.005 g of magnesium chloride hexahydrate, 0.375 g of sodium carboxymethylcellulose, 4.000 g of propylene glycol, 0.100 g of methylparaben, 0.010 g of propylparaben, and purified water to a total volume of 100.00 mL. The RM-GIC samples were composed of GC Fuji II LC RM-GIC in shade A2, with flat, smooth, and regular surfaces. A total of 80 RM-GIC specimens were measured to determine their baseline surface microhardness (33,34).

A Vickers microhardness tester (HV-1000 LG - Korea) was used to perform the hardness test. Surface mi-crohardness was measured using the Vickers test, expressed in kg/mm², which is calculated by dividing the applied load by the surface area of the indentation. The standard test method for material hardness using microindentation was applied, with each indentation spaced 0.5 to 1 mm apart and a dwell time of 15 seconds. Three measurements were taken on each sample before and after the application of fluoride and saliva solution in the experimental and control groups, respectively. These three measurements were averaged to obtain a final value equivalent to the mean microhardness.

After the initial measurement, samples were stored in an oven at 37°C for 24 hours, then washed and dried. Fluoride gels were applied to experimental groups 1 and 2 for 4 minutes (21,35), while fluoride varnish was applied to experimental group 3 for 60 seconds (29). The experimental groups were composed as follows: Group 1 consisted of 20 RM-GIC specimens immersed in 2% neutral sodium fluoride (Maquira Indústria de Produtos Odontológicos S.A., Maringá, Brazil); Group 2 included 20 RM-GIC specimens immersed in 1.23% acidulated phosphate fluoride (Laboratorios EUFAR S.A., Bogotá, Colombia); Group 3 contained 20 RM-GIC specimens immersed in 0.1% fluoride varnish (7700 ppm F) (Ivoclar ©, Vivadent, Schaan, Liechtenstein); and Group 4, the control group, included 20 RM-GIC specimens immersed in artificial saliva (Salival, Laboratorios Unidos S.A., Lima, Peru). Each group of samples was then placed in plastic containers with 20 mL of deionized water and stored at 37°C for 1 day. Finally, the surface microhardness was measured a second time.

-Statistical analysis

This study employed the non-parametric Kruskal-Wallis test to compare the overall hypothesis across the four groups. The Mann-Whitney test was used to assess microhardness mean differences between pairwise experimental and control groups, while the Wilcoxon paired test evaluated differences in pre- and post-treatment measures. All tests were conducted with a significance level of α = 0.05 using the software R v.4.4.0 with the packages ‘readxl’ (for reading spreadsheets), ‘tidyr’ (for managing and organizing data), ‘ggplot2’, ‘rbase’ and ‘ggpubr’ (for performing statistical analyses and plotting results). These non-parametric tests were selected after proper evaluation of the sample distribution using the Shapiro-Wilk test.

## Results

-Baseline microhardness of RM-GIC

**Figure 2:**
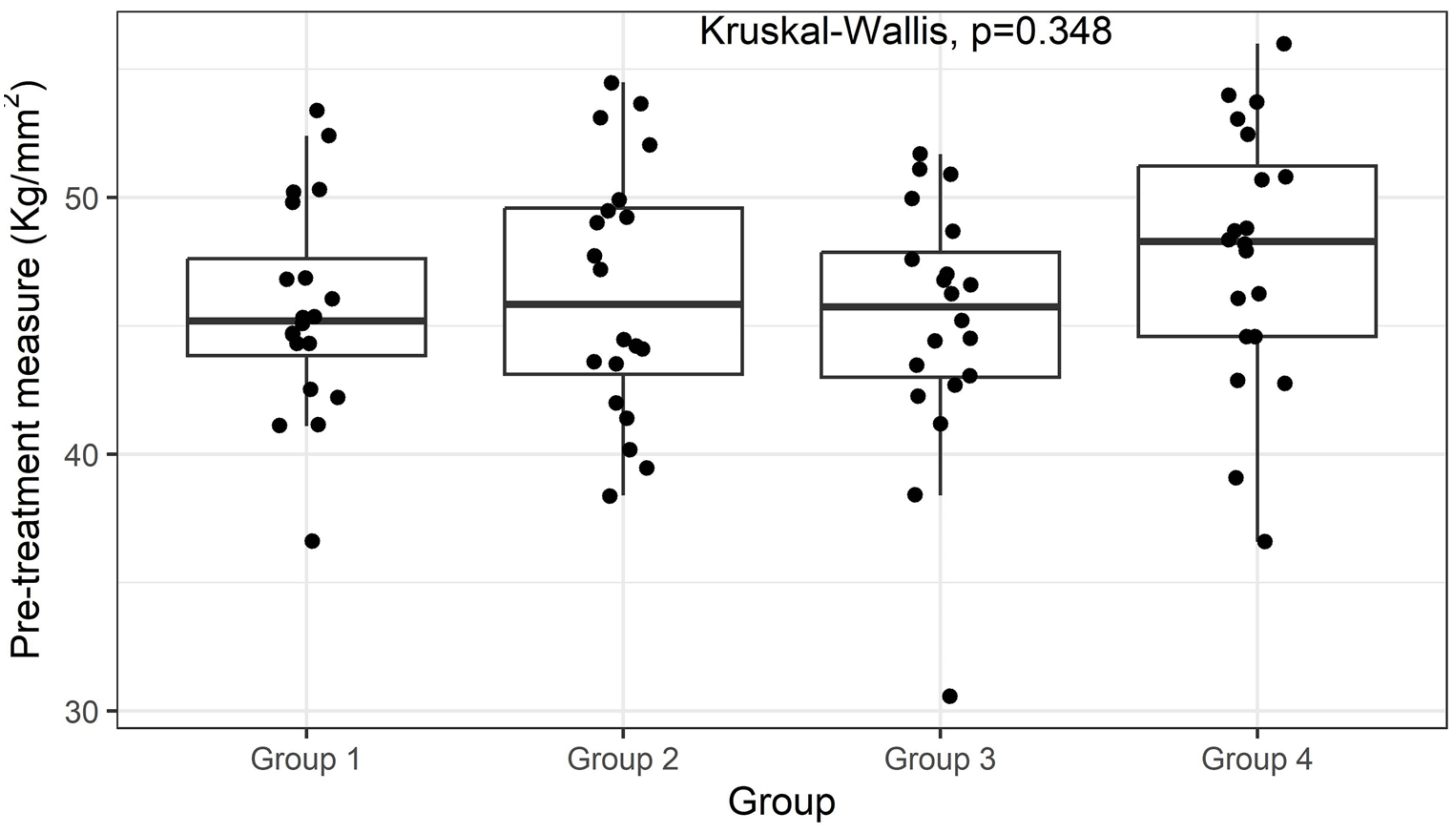
Pre-treatment microhardness of all RM-GIC discs. The boxplot illustrates data distribution across the experimental groups, with each disc represented by a point. The Kruskal-Wallis *p*-value indicates no significant differences in the micro-hardness of these discs before treatment.

At the start of the study, the average surface microhardness of the RM-GIC specimens was 46.2 ± 6.42 kg/mm² (median ± interquartile range-IQR), with no significant differences between the groups (Kruskal-Wallis *p-value* = 0.35, Fig. 2).

-Microhardness After Fluoride Exposure

**Figure 3:**
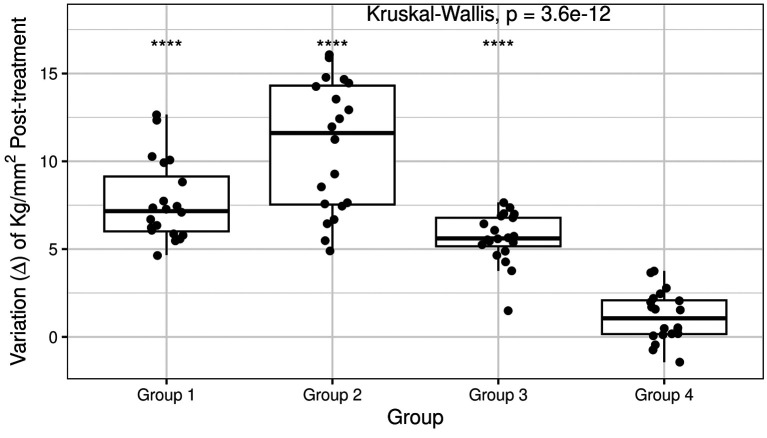
Post-treatment variation in RM-GIC microhardness. The boxplot shows data distribution across experimental groups, with each disc represented by a point. Group 1: 2% neutral sodium fluoride; Group 2: 1.23% acidulated phosphate fluoride; Group 3: 0.1% fluoride varnish (7700 ppm F); Group 4: artificial saliva (control). The Kruskal-Wallis *p*-value reflects significant dif-ferences in the effect of each treatment on RM-GIC microhardness. Asterisks indicate the Mann-Whitney two-group comparison between each treatment group and the control group (Group 4). ****: *p*<0.0001.

After treating each group of RM-GIC discs with different fluoride products, significant differences were ob-served in the variation of microhardness (difference between post-treatment and pre-treatment values) among the groups (Kruskal-Wallis *p-value* = 3.6x10¹², Fig. 3).

The average surface microhardness of RM-GIC in Group 1 decreased after applying 2% neutral sodium fluoride (*p* < 0.01,  Table 1). Similarly, the average microhardness in Group 2 dropped after exposure to 1.23% acidulated phosphate fluoride (*p* < 0.01,  Table 1). In Group 3, the application of 0.1% fluoride varnish (7700 ppm F) decreased microhardness to 42.7 ± 4.65 kg/mm² following (*p* < 0.05,  Table 1). The average surface micro-hardness for the control group (Group 4) did not show statistically significant alterations (*p* = 0.208,  Table 1).

**Figure 4:**
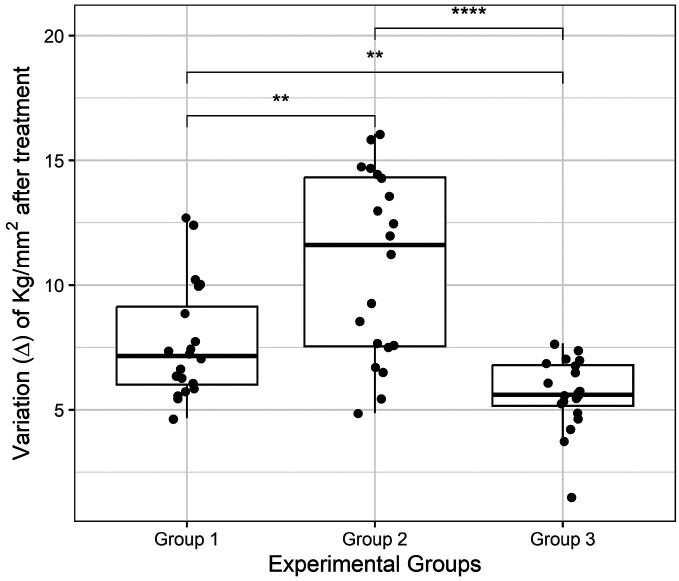
Post-treatment variation between experimental groups. Following the observation that all experimental groups were significantly different from the control group, we explored the differences between the experimental groups. The boxplot displays data distribution by experimental design groups, with each disc represented by a point. Group 1 (2% neutral sodium fluoride), Group 2 (1.23% acidulated phosphate fluoride), Group 3 (0.1% fluoride varnish, 7700 ppm F). The Mann-Whitney two-group test shows differences between pairwise experimental groups. **: *p*<0.01; ****: *p*<0.0001.

Figure 3 highlights that all treatment groups exhibited a reduction in microhardness, with Groups 1 and 2 showing the greatest impact on glass ionomers (Fig. 4). In particular, Group 2, which was exposed to 1.23% acidulated phosphate fluoride, demonstrated the most significant decrease in microhardness ( Table 1).

Overall, the average microhardness values in the treatment groups were lower in the final phase compared to the initial phase, indicating a reduction following fluoride application. Naturally, the control group showed no statistically significant changes in microhardness over time (*p*=0.208, ΔKg/mm2 ranging -1.44 to 3.76), suggesting no adverse effect in the absence of fluoride exposure (Fig. 3).

## Discussion

Based on the results obtained, the null hypothesis was rejected, as significant differences were observed in the surface microhardness of the RM-GIC when immersed in different fluoride solutions.

Dental restorative materials release fluoride, influencing caries formation by promoting repair or arrest of lesions. Fluoride helps reduce dental substrate demineralization and promotes remineralization of hard tissues (11). Commercial fluoride gels and varnishes can increase the fluoride content of glass ionomer cements (GICs), forming fluoride reservoirs. However, the high reactivity of these fluorinated agents can negatively affect the properties of certain aesthetic restorative materials (9).

Glass ionomer cements (GIC) are widely accepted in preventive dentistry due to their cariostatic properties, fluoride release, biocompatibility, aesthetics, and good adhesion to enamel and dentin (9,11,12). Resin-modified glass ionomer cements (RM-GIC) were later developed, offering enhanced clinical performance due to the presence of polymerizable functional groups, allowing for faster hardening and increased resistance to moisture sensitivity and lower mechanical strength (13,36). This study applied 1.23% acidulated phosphate fluoride gel (pH 3.5) and 2% sodium fluoride (NaF) gel (pH 7) for 4 minutes, as recommended by the American Dental Association for professional topical fluoride applications (9,37). Fluoride varnishes, known for their high fluoride content and extended durability of about 12 hours on tooth surfaces, are often used to address erosion and abrasion issues due to the deposition of calcium fluoride (CaF2) (29).

The hardness of a material is related to various mechanical properties, including its resistance to abrasion or wear, a property with clinical relevance (9,38-40). This study used the Vickers microhardness test to assess the hardness of dental restorative materials.

The topical agents used—1.23% APF, 2% NaF, and 0.1% fluoride varnish—produced a statistically significant decrease in microhardness values for RM-GIC, except in the control group, thus rejecting the null hypothesis. According to studies by Wilde *et al*. (13) and De Witte *et al*. (41), the reduction in microhardness is attributed to the overload of fluoride caused by topical applications, resulting in increased surface roughness, porosity, and material degradation over time (42). Therefore, it is crucial to assess the impact of commercial fluoride gels and varnishes on the mechanical and aesthetic properties of restorative materials.

The disintegration of RM-GIC occurs through selective attack on residual glass particles, forming ionic com-plexes between carboxylic groups and metal ions. The pH of the environment also plays a role, as acidic conditions can further reduce microhardness (13).

One topical agent, 1.23% APF, contains phosphoric acid, which enhances fluoride uptake by etching the enamel. However, previous research has demonstrated that phosphoric acid can significantly alter the surface morphology of restorative materials, negatively impacting microhardness and other physical properties (9,21,22). Studies have shown that 1.23% APF causes erosion on restorative materials, while 2% NaF gel, which lacks acidic components, has less effect (13,40,43).

In this study, 1.23% APF produced the most significant decrease in RM-GIC microhardness, while 2% NaF had a lesser effect. These findings align with previous studies by Gill *et al*. (9), Setty *et al*. (44), and El-Badrawy *et al*. (45). The reduced microhardness in RM-GIC may be explained by the presence of photopolymerizable monomers, which undergo an acid-base reaction along with a secondary light-activated or chemically-activated reaction (45). In contrast, conventional GICs undergo only the acid-base reaction, resulting in a more substantial decrease in microhardness (9,45). While Gill *et al*. (9) observed no significant reduction in GIC or RM-GIC microhardness with 2% NaF gel, the current study found a significant decrease in RM-GIC microhardness after applying the same gel (Fuji LC II). This discrepancy may be due to differences in methodology, such as storage conditions (artificial saliva versus distilled water) and the method and duration of fluoride application.

Fluoride varnishes release fluorapatite, providing additional protection against caries. However, the current study observed a noTable decrease in RM-GIC microhardness after 60 seconds of exposure to fluoride varnish. Further research is needed to explore the effects of fluoride varnishes on various restorative materials, including newer materials like zirconia-reinforced GICs, which have shown improved physical properties (38). RM-GIC and topical fluorides like APF, NaF, and varnishes are commonly used in dentistry to prevent caries. However, it is crucial to understand the impact of different fluoride agents on restorations and restorative materials, as studies indicate that fluoride recharges in RM-GIC can lead to adverse effects, including fractures, discoloration, roughness, and porosity over time.

This study presents some strengths and limitations. Among the strengths, the study follows a rigorous *in vitro* design with clear experimental controls, which allows for detailed observation of how fluoride agents impact the microhardness of RM-GIC. The use of multiple fluoride treatments (neutral sodium fluoride, acidulated phosphate fluoride, and fluoride varnish) provides a broad understanding of their effects on restorative materials. Moreover, the use of standardized ISO specifications for specimen preparation enhances the reproducibility of the study.

However, the study’s limitations primarily stem from its *in vitro* nature, which may not fully represent clinical conditions. The absence of real-world factors like patient saliva composition, diet, and oral hygiene limits the direct applicability of the findings to clinical practice. Additionally, while the study focuses on microhardness, it does not explore other important mechanical properties such as wear resistance and fracture toughness, which could provide a more comprehensive assessment of material performance under fluoride treatment. Further clinical trials are necessary to validate these *in vitro* findings.

## Conclusions

Applying 2% neutral sodium fluoride, 1.23% acidulated phosphate fluoride, and 0.1% fluoride varnish (7700 ppm F) to RM-GIC results in decreased surface microhardness. The most significant reduction was observed in Group 2 (1.23% acidulated phosphate fluoride), followed by Group 1 (2% neutral sodium fluoride). Therefore, it is essential to consider the type of fluoride and its potential effects on ionomeric restorative materials during preventive treatments.

## Figures and Tables

**Table 1:** Comparison of the surface microhardness of RM-GIC discs pre-treatment and post-treatment according to their re-spective groups.

Groups	N	Wilcoxon p-value	Minimum	Maximum	Mean	SD	Median	IQR
Group 1(2% neutral sodium fluoride)	Pre-treatment (n=20)	p=0.002	36.6	53.4	45.7	4.09	45.2	3.78
	Post-treatment (n=20)		33.5	46.8	41.1	3.81	42.5	4.9
Group 2(1.23% acidulated phosphate fluoride)	Pre-treatment (n=20)	p=0.00006	38.4	54.5	46.4	4.92	45.8	6.48
	Post-treatment (n=20)		29.7	49.5	38.7	4.42	39.2	4.67
Group 3(0.1% fluoride varnish)	Pre-treatment (n=20)	p=0.036	30.6	51.7	45.1	4.91	45.8	4.88
	Post-treatment (n=20)		32.3	49.5	42.6	4.28	42.7	4.65
Group 4(control)	Pre-treatment (n=20)	p=0.208	36.6	56	47.8	5.06	48.3	6.62
	Post-treatment (n=20)		39.6	58.3	49.8	5.25	49.4	7.92

*p*-values were estimated using the Wilcoxon paired test. Bold *p*-values indicate groups with statistical differences between pre and after-treatment microhardness (*p*<0.05). SD:Standard Deviation; IQR: Interquartile Range.

## Data Availability

Data is contained within the article.

## References

[1] Hamilton IR (1990). Biochemical Effects of Fluoride on Oral Bacteria. J Dent Res.

[2] AlMatar D, AlSanousi S, Ahmed J, Saad Bin Qasim S (2023). The In-Vitro Effect of Silver and Zinc Oxide Nanoparticles on Fluoride Release and Microhardness of a Resin-Modified Glass Ionomer Cement. J Inorg Organomet Polym Mater.

[3] Mariano Mundim F, Rodrigues Cruvinel D, da Fonseca Roberti Garcia L, de Carvalho Panzeri Pires-de-Souza F (2014). Effect of Fluoride Solutions on Color and Surface Roughness of Dental Composites. Revista da Faculdade de Odontologia – UPF.

[4] Forss H (1993). Release of Fluoride and Other Elements from Light-Cured Glass Ionomers in Neutral and Acidic Conditions. J Dent Res.

[5] Cabello Malagón I, Cánovas Hernández B, Martínez Hernández E, Serna-Muñoz C, Pérez-Silva A, Ortiz-Ruiz AJ (2022). Analysis of the Porosity and Microhardness of Glass Ionomer Cements. Medziagotyra.

[6] Gunay A, Celenk S, Adiguzel O, Cangul S, Ozcan N, Cakmakoglu EE (2023). Comparison of Antibacterial Activity, Cytotoxicity, and Fluoride Release of Glass Ionomer Restorative Dental Cements in Dentistry. Med Sci Monit.

[7] Adresi Y, Kam Hepdeniz Ö, Demirel Üniversitesi S, Hekimliği Fakültesi D, Diş Tedavisi Anabilim Dalı R, İyonomer İçerikli Dört Farklı Restoratif Materyalin Yüzey Pürüzlülüklerinin Değerlendirilmesi C (2019). Cam İyonomer İçerikli Dört Farklı Restoratif Materyalin Yüzey Pürüzlülüklerinin Değerlendirilmesi. Süleyman Demirel Üniversitesi Sağlık Bilimleri Dergisi.

[8] Basting RT, Serra MC, Rodrigues AL (2002). In Situ Microhardness Evaluation of Glass-Ionomer/Composite Resin Hybrid Materials at Different Post-Irradiation Times. J Oral Rehabil.

[9] Gill NC, Pathak A (2010). Comparative Evaluation of the Effect of Topical Fluorides on the Microhardness of Various Restorative Materials: An in Vitro Study. J Indian Soc Pedod Prev Dent.

[10] P UN, Kishore G (2005). Glass Ionomer Cement - The Different Generations. Trends Biomater Artif Organs.

[11] Wiegand A, Buchalla W, Attin T (2007). Review on Fluoride-Releasing Restorative Materials--Fluoride Release and Uptake Characteristics, Antibacterial Activity and Influence on Caries Formation. Dent Mater.

[12] Moberg M, Brewster J, Nicholson J, Roberts H (2019). Physical Property Investigation of Contemporary Glass Ionomer and Resin-Modified Glass Ionomer Restorative Materials. Clin Oral Investig.

[13] Wilde MGK, Delfino CS, Sassi JF, Garcia PPNS, Palma-Dibb RG (2006). Influence of 0.05% Sodium Fluoride Solutions on Microhardness of Resin-Modified Glass Ionomer Cements. J Mater Sci Mater Med.

[14] Hadi MR (2020). Effect of Increased Fluoride Contents on Fluoride Release from Glass Ionomer Cements. Systematic Review Pharmacy.

[15] Okada K, Tosaki S, Hirota K, Hume WR (2001). Surface Hardness Change of Restorative Filling Materials Stored in Saliva. Dent Mater.

[16] Diaz-Arnold A, Dw W, Swift EJ (1995). Topical Fluoride and Glass Ionomer Microhardness. Am J Dent.

[17] Akwan YE, Paramita AL, Rahmitasari F (2022). Shear Bond Strength Fissure Sealant Based on Glass Ionomer after Topical Fluor Application: A Comparison between Sodium Fluoride and Acidulated Phosphate Fluoride. Odonto Dental Journal.

[18] Hatibovic-Kofman S, Koch G, Ekstrand J (1997). Glass Ionomer Materials as a Rechargeable Fluoride-Release System. Int J Paediatr Dent.

[19] Suljak JP, Hatibovic-Kofman S (1996). A Fluoride Release-Adsorption-Release System Applied to Fluoride-Releasing Restorative Materials. Quintessence Int.

[20] García-Godoy F, García-Godoy A, García-Godoy F (2003). Effect of APF Minute-Foam on the Surface Roughness, Hardness, and Micromorphology of High-Viscosity Glass Ionomers. J Dent Child (Chic).

[21] Yip HK, Lam WTC, Smales RJ (1999). Fluoride Release, Weight Loss and Erosive Wear of Modern Aesthetic Restoratives. Br Dent J.

[22] Yap AUJ, Mok BYY (2002). Effects of Professionally Applied Topical Fluorides on Surface Hardness of Composite-Based Restoratives. Oper Dent.

[23] Bezerra Wanderley Lima R, Karina Maciel Andrade Fábia Danielle Sales da Cunha Medeiros Silva A, Marques Duarte R Revista Brasileira de Odontologia Influência Da Aplicação Tópica de Géis de Flúor Na Rugosidade Superficial de Cimentos de Ionômero..

[24] Khosla S, Verma V, Markan S (2018). Comparison of Surface Roughness of Two Restorative Materials after the Application of Topical Fluorides. International Healthcare Research Journal.

[25] Krithikadatta J, Gopikrishna V, Datta M (2014). CRIS Guidelines (Checklist for Reporting In-Vitro Studies): A Concept Note on the Need for Standardized Guidelines for Improving Quality and Transparency in Reporting in-Vitro Studies in Experimental Dental Research. J Conserv Dent.

[26] Thompson SO, Griffin GD, Meyer N, Pelaez M (2017). Effect of Smokeless Tobacco on Surface Roughness of Dental Restorations. US Army Med Dep J.

[27] Bayrak S, Sen Tunc E, Tuloglu N, Ceylan G (2011). Effects of Self-etch Adhesives Used as Surface Coating Agents on Microleakage of Conventional and Resin Modified Glass Ionomer Cements. Materials Research Innovations.

[28] Sungurtekin-Ekci E, Ozdemir-Ozenen D, Duman S, Acuner IC, Sandalli N (2015). Antibacterial Surface Properties of Various Fluoride-Releasing Restorative Materials in Vitro. J Appl Biomater Funct Mater.

[29] Moharramkhani F, Omrani LR, Abbasi M, Kharrazifard MJ, Ahmadi E (2021). Effect of Fluoride Varnish on Glass Ionomer Microhardness Changes in Endogenous Acid Erosion Challenge. Biomater Investig Dent.

[30] Özveren N, Baltaci E, Batur Kara S (2021). Effect of Mouthrinses on Water Sorption and Solubility of Flouride-Releasing Restorative Materials. Bezmialem Science.

[31] Schlafer S, Bornmann T, Paris S, Göstemeyer G (2021). The Impact of Glass Ionomer Cement and Composite Resin on Microscale PH in Cariogenic Biofilms and Demineralization of Dental Tissues. Dent Mater.

[32] Warren DP, Colescott TD, Henson HA, Powers JM (2002). Effects of Four Prophylaxis Pastes on Surface Roughness of a Composite, a Hybrid Ionomer, and a Compomer Restorative Material. J Esthet Restor Dent.

[33] Bilić-Prcić M, Šalinović I, Gurgan S, Vural UK, Krmek SJ, Miletić I (2021). Effects of Incorporation of Marine Derived Hydroxyapatite on the Microhardness, Surface Roughness, and Fluoride Release of Two Glass-Ionomer Cements. Applied Sciences.

[34] Dionysopoulos D, Tolidis K, Sfeikos T, Karanasiou C, Parisi X (2017). Evaluation of Surface Microhardness and Abrasion Resistance of Two Dental Glass Ionomer Cement Materials after Radiant Heat Treatment. Advances in Materials Science and Engineering.

[35] Kula K, McKinney JE, Kula TJ (1997). Effects of Daily Topical Fluoride Gels on Resin Composite Degradation and Wear. Dent Mater.

[36] (1986). A guide to the use of fluorides for the prevention of dental caries. J Am Dent Assoc.

[37] Anusavice KJ (2003). Dental Ceramics. In "Phillips" Science of Dental Materials'.

[38] Bethapudy DR, Bhat C, Lakade L, Chaudhary S, Kunte S, Patil S (2022). Comparative Evaluation of Water Sorption, Solubility, and Microhardness of Zirconia-Reinforced Glass Ionomer, Resin-Modified Glass Ionomer, and Type IX Glass Ionomer Restorative Materials: An In Vitro Study. Int J Clin Pediatr Dent.

[39] Alshehri TD, Kotha SB, Abed FM, Barry MJ, Alasmari A, Mallineni SK (2022). Effect of the Addition of Varying Concentrations of Silver Nanoparticles on the Fluoride Uptake and Recharge of Glass Ionomer Cement. Nanomaterials (Basel).

[40] Abate PF, Bertacchini SM, Garcia-Godoy F, Macchi RL (2001). Barcoll Hardness of Dental Materials Treated with an APF Foam. J Clin Pediatr Dent.

[41] De Witte AMJC, De Maeyer EAP, Verbeeck RMH (2003). Surface Roughening of Glass Ionomer Cements by Neutral NaF Solutions. Biomaterials.

[42] Diaz-Arnold AM, Holmes DC, Wistrom DW, Swift EJ (1995). Short-Term Fluoride Release/Uptake of Glass Ionomer Restoratives. Dent Mater.

[43] K K, EL W, TJ K (1996). Effect of 1- and 4-Minute Treatments of Topical Fluorides on a Composite Resin. Pediatr Dent.

[44] Setty JV, Singh S, Subba Reddy VV (2003). Comparison of the effect of topical fluorides on the commercially available conventional glass ionomers, resin modified glass ionomers and polyacid modified composite resins--an in vitro study. J Indian Soc Pedod Prev Dent.

[45] El-Badrawy W, McComb D (1998). Effect of Home-Use Fluoride Gels on Resin-Modified Glass-Ionomer Cements. Oper Dent.

